# A Comprehensive, Multidisciplinary, Personalized, Lifestyle Intervention Program Is Associated with Increased Leukocyte Telomere Length in Children and Adolescents with Overweight and Obesity

**DOI:** 10.3390/nu13082682

**Published:** 2021-08-02

**Authors:** George Paltoglou, Christina Raftopoulou, Nicolas C. Nicolaides, Sofia M. Genitsaridi, Sofia I. Karampatsou, Marina Papadopoulou, Penio Kassari, Evangelia Charmandari

**Affiliations:** 1 Division of Endocrinology, Metabolism and Diabetes, First Department of Pediatrics, National and Kapodistrian University of Athens Medical School, ‘Aghia Sophia’ Children’s Hospital, 11527 Athens, Greece; gpaltoglou@gmail.com (G.P.); nicolaidesnc@gmail.com (N.C.N.); sgenitsaridi@gmail.com (S.M.G.); karampatsousi@gmail.com (S.I.K.); marinageorpap@gmail.com (M.P.); peniokassari@gmail.com (P.K.); 2 Division of Endocrinology and Metabolism, Center for Clinical, Experimental Surgery and Translational Research, Biomedical Research Foundation of the Academy of Athens, 11527 Athens, Greece; xraftopoulou@yahoo.gr

**Keywords:** overweight, obesity, leukocyte telomere length, childhood, adolescence

## Abstract

Leucocyte telomere length (LTL) is a robust marker of biological aging and is associated with obesity and cardiometabolic risk factors in childhood and adolescence. We investigated the effect of a structured, comprehensive, multidisciplinary, personalized, lifestyle intervention program of healthy diet and physical exercise on LTL in 508 children and adolescents (239 males, 269 females; 282 prepubertal, 226 pubertal), aged 10.14 ± 0.13 years. Participants were classified as obese (*n* = 267, 52.6%), overweight (*n* = 174, 34.2%), or of normal BMI (*n* = 67, 13.2%) according to the International Obesity Task Force (IOTF) cutoff points and were studied prospectively for one year. We demonstrated that LTL increased significantly after 1 year of the lifestyle interventions, irrespective of gender, pubertal status, or body mass index (BMI). Waist circumference was the best negative predictor of LTL at initial assessment. The implementation of the lifestyle interventions also resulted in a significant improvement in clinical (BMI, BMI z-score and waist to height ratio) and body composition indices of obesity, inflammatory markers, hepatic enzymes, glycated hemoglobin (HbA1C), quantitative insulin sensitivity check index (QUICKI), and lipid profile in all participants. These findings indicate that the increased LTL may be associated with a more favorable metabolic profile and decreased morbidity later in life.

## 1. Introduction

Telomeres in humans are tandem repeats of hexameric (5′-TTAGGG-3′) nucleotide sequences at the ends of linear chromosomes, which represent part of a complex ribonucleoprotein structure that caps the end of each chromosome and erodes with each cell division [1]. This cap protects the chromosome from nucleolytic degradation and prevents their recognition as sites of DNA damage [1]. Telomere length preservation or the dysfunction of end-capping was previously linked to genomic instability and ageing [2]. Furthermore, cumulative exposure to agents that result in DNA damage, such as oxidative, inflammatory, and other forms of biological stress lead to telomere attrition [3]. Leucocyte telomere length (LTL) is an established biomarker of biological aging (telomeres shorten in an age-dependent manner), that varies considerably among individuals and is associated with cardiovascular risk factors, such as essential hypertension, insulin resistance and diabetes mellitus type 2, as well as pathological processes associated with oxidative stress [4,5,6,7].

Obesity in childhood or adolescence represents one of the greatest challenges in public health of the 21st century, and the term ‘epidemic’ is commonly used to indicate the rapid increase in its prevalence during the last four decades [8]. In 2015, a total of 107.7 million children and 603.7 million adults had obesity, and the prevalence of obesity increases more rapidly in children than in adults in many countries [9]. The prevalence of overweight and obesity in childhood and adolescence in Greece exceeds 30–35%, which is among the highest rates in Europe [10]. Obesity in childhood and adolescence leads to obesity in adulthood and is associated with significant morbidity and mortality, as well as a significant increase in public health costs [11]. Complications of obesity include insulin resistance, glucose intolerance, diabetes mellitus type 2, dyslipidemia, hypertension, endothelial dysfunction, early onset atherosclerotic cardiovascular disease, hypogonadism, orthopedic problems, fatty liver disease, cholecystitis, social stigmatization, and increased incidence of certain malignancies [8]. Therefore, it is imperative that the prevention and management of overweight and obesity should commence in childhood to ensure improved health in adulthood, as well as reduced medical costs owing to complications of obesity. To address the epidemic of childhood obesity in our country, we developed novel e-Health applications that are expected to play an important role in the prevention and management of overweight and obesity in children and adolescents [12,13]. In addition, we demonstrated that a structured, comprehensive, multidisciplinary intervention program that provides detailed and individualized advice on diet and exercise are effective at reducing the prevalence of overweight and obesity in childhood and adolescence [12,13,14].

Several conditions that arise as complications of obesity are associated with decreased LTL, and this association is evident even in childhood [6,15,16,17,18,19]. Although few in number, these human population studies investigated the association of LTL and obesity in children and adolescents and provided clues that weight loss via a structured multidisciplinary program might increase LTL, while greater LTL could potentially predict a better weight loss response to an intervention program in overweight/obese male adolescents [16]. Furthermore, there is evidence that obesity-associated LTL differences in gender might follow the sexual dimorphic pattern of longevity [7,20,21]. The biology of cardiovascular aging differs between men and women, with men with shorter LTL demonstrating more elevated markers of cardiovascular aging compared with women [20].

The aim of our prospective study was to investigate the effect of a 12-months’ structured, comprehensive, multidisciplinary, personalized lifestyle intervention program of healthy diet and physical exercise on leukocyte telomere length in children and adolescents. The primary aim was to determine differences in LTL before and after the 12-months’ structured intervention program, according to body mass index (BMI), gender and pubertal status, and to define potential factors that influence these changes. Our study included a larger number of participants than previously reported, which allowed us to also investigate the possible role of gender and/or puberty on the association of BMI and LTL.

## 2. Materials and Methods

### 2.1. Study Design

This was a prospective study of a cohort of participants attending the Out-patient Clinic for the Prevention and Management of Overweight and Obesity in Childhood and Adolescence, Division of Endocrinology, Metabolism and Diabetes, First Department of Pediatrics, National and Kapodistrian University of Athens Medical School, ‘Aghia Sophia’ Children’s Hospital, who participated in a structured, comprehensive, multidisciplinary, and personalized lifestyle intervention program of diet and exercise for 12 months. The study included data of the initial and annual follow-up visit. The study was conducted in accordance with the Declaration of Helsinki and was approved by the Committee on the Ethics of Human Research of ‘Aghia Sophia’ Children’s Hospital (Approval Number: EB-PASCH-MoM: 28/11/2013, Re: 10290-14/05/2013). The aims and the procedures of the study were fully disclosed to the parents or legal guardians of the participants. Written informed consent was obtained in all cases by a parent/guardian, and assent was given by children older than 7 years. There was no difference to the care given to patients that did not opt to participate or did not complete the study. 

### 2.2. Participants

Five hundred and eight (n_total_ = 508; 239 males, 269 females) children and adolescents, aged 10.14 ± 0.13 years (mean ± SE), consecutively attending our Out-patient Clinic for the Prevention and Management of Overweight and Obesity in Childhood and Adolescence were recruited to participate in the study. All participants were evaluated by a Pediatrician, Pediatric Endocrinologist, Pediatric Dietician, a professional fitness Personal Trainer and—when necessary—a Pediatric Clinical Psychologist, and entered a personalized, comprehensive, multidisciplinary management intervention program that provided personalized advice and guidance on healthy diet and physical exercise to participants and their families [13,14]. 

### 2.3. Study Protocol

#### 2.3.1. Initial Assessment

All participants were admitted to the Endocrine Unit early in the morning on the day of the first visit, and a single trained observer obtained a detailed medical history and clinical examination, including Tanner pubertal assessment and standard anthropometric measurements (weight, height, waist circumference, hip circumference). Detailed hematologic, biochemical, and endocrinologic investigations, as well as body composition analysis and LTL measurement, were performed at 08:00 h following a 12-h fast. Pediatric Dietician, who advised them to eat regularly throughout the day and have three main meals (breakfast, lunch, and dinner) and two healthy snacks (fruits, vegetables) at mid-morning and mid-afternoon. The composition of main meals was suggested according to the 2010 USDA guidelines and the guidelines proposed by the National Nutritional Guide for Infants, Children, and Adolescents [22]. In addition, they were evaluated by a professional fitness Personal Trainer, and their activities and hobbies throughout the week were recorded. Subsequently, the personal trainer discussed the child’s interests with the family to identify suitable sport activities.

#### 2.3.2. Intervention

At initial assessment participants were classified as obese (*n* = 267, 52.6%), over-weight (*n* = 174, 34.2%) or of normal BMI (*n* = 67, 13.2%) according to the International Obesity Task Force (IOTF) cutoff points [23,24], and according to Tanner criteria as pre-pubertal (*n* = 282) and pubertal (*n* = 226). The participants were studied prospectively for one year with scheduled appointments according to a standardized follow-up protocol [10,12,13,14]. Subjects with obesity were followed up every month, those with overweight every 2 months and those with normal-BMI every 3 months. Upon each follow-up visit, all anthropometric measurements (weight, height, waist circumference, hip circumference) were determined, clinical examination and Tanner pubertal assessment were performed, a 24-h diet recall was recorded, and all the goals set at previous sessions were discussed in detail. Subsequently, all participants had their 24-h recall of their diet evaluated based on the USDA method by the Pediatric Dietitian and the Pediatrician, to minimize the reduced validity of 24-h in school aged children, and dietary counseling was provided [23,24]. 

#### 2.3.3. Annual Follow-Up Visit

All participants were admitted to the Endocrine Unit early in the morning on the day of the annual follow-up visit, and a detailed medical history and clinical examination, including Tanner pubertal assessment and standard anthropometric measurements (weight, height, waist circumference, hip circumference) were obtained by a single trained observer. Following the clinical assessment detailed hematologic, biochemical, and endocrinologic investigations, as well as body composition analysis and LTL measurement, were performed, at 08:00 h following a 12-h fast. Good compliance for the study purposes was evaluated at the annual follow-up appointment and was defined as 100% compliance with the follow-up visits and the above-mentioned separate evaluations of the multidisciplinary team. In total, 407 (80.1%) of the initially 508 enrolled participants completed all the required follow-up visits in the 12 months of the Multidisciplinary Structured Lifestyle Intervention Program [189 males, 218 females; aged 11.23 ± 0.15 years (mean ± SE)]; these were included in the subjects with “good compliance” group and their data were further analyzed. All participants included in the study self-reported good adherence with the advice given on diet and exercise. The clinical characteristics of subjects with “good compliance” are summarized in Table 1A.

### 2.4. Anthropometric and Body Composition Parameters

Body weight was measured in light clothing and without shoes using the same scale for all participants (Seca GmbH & Co. KG., Hamburg, Germany). Standing height was also measured without shoes using a stadiometer (Holtain Limited, Crymych–Dyfed, UK). Body mass index (BMI) was calculated as body weight (in kg) divided by height (in m) squared and expressed in kg/m^2^. Subsequently, BMI z-score was calculated according to the Greek standard growth charts [25]. In addition, World Health Organization (WHO) STEPwise approach to Surveillance (STEPS) protocol was used for waist and hip circumferences measurements by employing the same stretch-resistant tape (Seca GmbH & Co. KG., Hamburg, Germany). Waist circumference was measured, with the participant standing, in the horizontal plane midway between the lowest rib and the iliac crest at the end of a normal expiration, while hip circumference was also measured in the horizontal plane at the level of maximum circumference of hips and buttocks. Blood pressure was determined by a sphygmomanometer with an appropriate cuff for the age of the participant (Comfort 20/40, Visomat, Parapharm, Metamorphosi, Attiki, Greece) [14]. Furthermore, each participant underwent bioelectrical impedance analysis (BIA) (TANITA MC-780U Multi Frequency Segmental Body Composition Analyzer, Amsterdam, The Netherlands). Blood samples for baseline hematologic, biochemical, and endocrinologic investigations, and LTL measurement were obtained at 08:00 h after a 12-h overnight fast. Samples were centrifuged and separated immediately after collection and were stored at −80 °C until assayed.

### 2.5. Assays

Hematologic investigations were performed in the ADVIA 2110i analyzer (Roche Diagnostics GmbH, Mannheim, Germany). The concentrations of glucose, total cholesterol, triglycerides and high-density lipoprotein cholesterol were determined in the ADVIA 1800 Siemens analyzer (Siemens Healthcare Diagnostics, Tarrytown, NY, USA). Apolipoprotein A1, B and lipoprotein (a) concentrations were determined by means of latex particle-enhanced immunonephelometric assays on the BN ProSpec nephelometer (Dade Behring, Siemens Healthcare Diagnostics, Liederbach, Germany). Hemoglobin A1C was measured on an automated glycohemoglobin analyzer HA-8160 (Arkray, Kyoto, Japan) by employing reversed-phase cation exchange high performance liquid chromatography. Insulin resistance was assessed using the homeostasis model assessment (HOMA-IR) method as follows: HOMA-IR = [fasting glucose (mg/dL) × fasting insulin (mU/L)]/405. In addition quantitative insulin sensitivity check index (QUICKI) was calculated using the following formula: QUICKI= 1/[log(fasting insulin µU/mL) + log(fasting glucose mg/dL)].

Follicle-stimulating hormone (FSH), luteinizing hormone (LH), estradiol, ferritin, and insulin concentrations were measured in an automated electrochemiluminescense immunoassays analyzer (Cobas e411, Roche Diagnostics GmbH, Mannheim, Germany). Thyroid-stimulating hormone (TSH), free thyroxine, antithyroid peroxidase antibodies, anti-thyroglobulin antibodies), adrenocorticotropin (ACTH), cortisol, androstenedione, testosterone, dehydroepiandrosterone (DHEA), dehydroepiandrosterone sulfate (DHEAS), insulin-like growth factor-I (IGF-I), insulin-like growth factor binding protein-3 (IGFBP-3), and high-sensitivity C-reactive protein (hsCRP) concentrations were measured by chemiluminescence immunoassays on an IMMULITE 2000 immunoassay system (Siemens Healthcare Diagnostics Products Ltd., Surrey, UK). Total 25-hydroxyvitamin D concentration was measured by employing an automated electrochemiluminescence immunoassay on the Modular Analytics E170 analyzer (Roche Diagnostics, Basel, Switzerland). 

### 2.6. Leucocyte Telomere Length

Leucocyte telomere length (LTL) was determined by employing multiplex monochrome quantitative real-time polymerase chain reaction (PCR) as previously described [18,26]. In brief, QiaGenMaxiprep kit (Qiagen) was used for genomic DNA extraction from peripheral leucocytes. All quantitative real-time PCRs were performed in triplicates in a final volume of 12.5 μL. Briefly, 15 ng of template DNA were mixed with final concentrations of 1 × iTaq™ Universal SYBR^®^ Green Supermix (Bio-Rad Laboratories, Hercules, CA, USA), 900 nm of the primers telg and telc, and 500 nm of hbgd and hbgu as single-copy gene primers. All PCRs were performed in 96-well plates using the CFX real-time PCR detection system (Bio-Rad Laboratories). Five serial dilutions of DNA extracted from peripheral leucocytes of an adult female (reference sample) spanning 3.75–60 ng were run on each plate in duplicates. Two standard curves for each plate were generated from the reference sample DNA dilutions, using the CFX manager software (Bio-Rad Laboratories, Hercules, California, USA): the first standard curve for the telomere amplicon signal (T) and the second standard curve for the single-copy gene (haemoglobin) amplicon signal (S). The T/S ratios were then estimated for each sample as T, the copy number of the telomere template for experimental sample, divided by S, which represented the copy number of the single-copy gene template. Only T/S measurements with coefficient of variation of triplicate samples, of less than 15% were accepted for further analyses.

### 2.7. Statistical Analysis

All variables were normally distributed. Results are reported as mean ± SE. Statistical significance was set at *p* < *0.05*. All variables assessed at initial assessment and at 12 months’ follow-up were compared by employing repeated-measures analysis of variance test (ANOVA) with obese/overweight/normal-BMI, male/female and prepubertal/pubertal as between subjects’ factors. Significant main effects were revealed by Fischer’s (LSD) *post-hoc* test. Correlations of the studied variables were evaluated by the Pearson’s R coefficient. To investigate for potential predictors of LTL at initial assessment, at annual assessment and the respective change (Delta) of LTL, all taken as dependent variables, standard forward, stepwise multiple regression models were employed. In the first model that assessed metabolic syndrome parameters, systolic blood pressure (SBP), waist circumference, cholesterol, triglycerides, and high-density lipoprotein (HDL) were taken as independent variables. In the second model that assessed body composition parameters, fat percentage, fat mass, muscle mass, bone mass, fat free mass, total body water, and basal metabolic rate (BMR) were taken as independent variables. In the third model that assessed anthropometric parameters, weight, height, BMI, waist, hip circumference, waist to hip ratio and waist to height ratio measurements were taken as independent variables. In the fourth and final model that assessed glucose metabolism and insulin sensitivity parameters, glucose, insulin, glycated hemoglobin (HbA1C), homeostatic model assessment (HOMA), and quantitative insulin sensitivity check index (QUICKI) measurements were taken as independent variables.

LTL measurements were further retrospectively assessed in participants that were successful at weight loss via two categorical criteria. Participants were considered successful if the respective BMI z-score decreased more than 0.6 at 12 months follow-up or if the respective IOTF category (obese, overweight, normal-BMI) improved by one or more steps. All statistical analyses were performed with the Statistica 8 software (StatSoft, Tulsa, OK, USA).

## 3. Results

### 3.1. Leukocyte Telomere Length (LTL)

**Figure 1:**
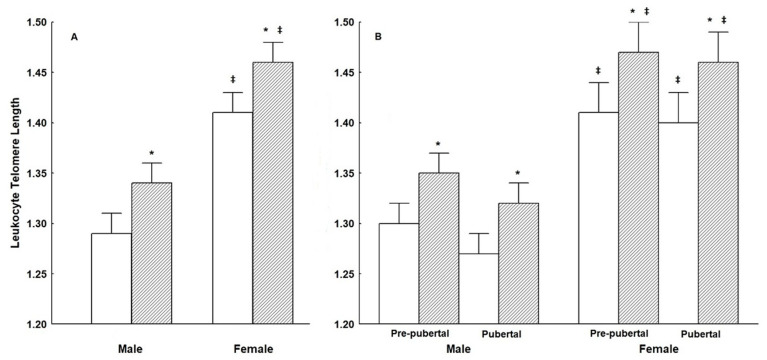
**Panel** (**A**): leukocyte telomere length (LTL) (mean ± SE) of male and female subjects with “good compliance” (*n* = 407), at initial assessment (white bars) and at 12 months (shaded bars). **Panel** (**B**): leukocyte telomere length (LTL) of male and female, prepubertal, and pubertal at initial assessment subjects with “good compliance” (*n* = 407), at initial assessment (white bars) and at 12 months (shaded bars). All measured variables were compared by employing repeated-measures ANOVA. Significant main effects were revealed by LSD post-hoc test. Statistical significance was set at (*p* < 0.05). * Indicates significant difference between initial assessment and the 12 months follow-up, time-points; ‡ Indicates significant difference from the Male group.

**Figure 2:**
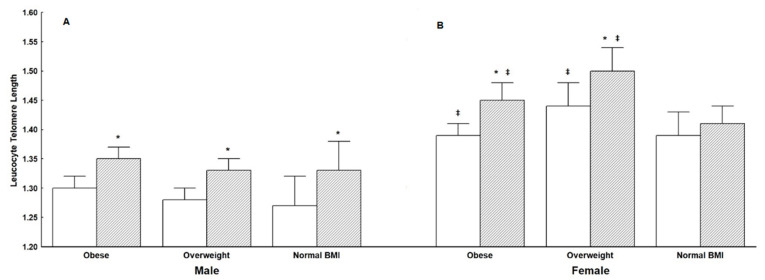
(**A**) Leukocyte telomere length (LTL) (mean ± SE) of male and female subjects with “good compliance” (*n* = 407), who were grouped as obese, overweight, or of normal-BMI at initial assessment (white bars) and same participants at 12 months (shaded bars). Panel (**B**): leukocyte telomere length (LTL) of male and female, prepubertal, and pubertal who were grouped as obese, overweight, or of normal-BMI at initial assessment (white bars) and at 12 months (shaded bars). All measured variables were compared by employing repeated-measures ANOVA. Significant main effects were revealed by the LSD posthoc test. Statistical significance was set at (*p* < 0.05). * Indicates significant difference between initial assessment and 12 months follow-up, time-points; ‡ Indicates significant difference from the Male group.

LTL increased significantly after the 1 year of lifestyle intervention program of healthy diet and physical exercise when all participants were considered (*p* < 0.01) (Table 2G). This significant increase in LTL following the reduction of BMI persisted when data were analyzed according to gender (as illustrated in Figure 1A) and Tanner pubertal status at initial assessment (as illustrated in Figure 1B). Furthermore, the significant post-intervention increase in LTL was present in obese, overweight, and normal-BMI male participants (as illustrated in Figure 2A), and obese and overweight female participants (as illustrated in Figure 2B).

Table 3 illustrates the LTL measurements of participants with “good compliance” (*n* = 407), according to the criterion of success at weight loss. Participants were considered successful at losing weight if their respective BMI z-score decreased > 0.6 at 12 months follow-up (Criterion A) or if the respective IOTF category (obese, overweight, normal-BMI) improved by one or more categories (Criterion B). LTL increased in both the successful and not successful at losing weight participants, irrespective of the criterion used (*p* < 0.01).

### 3.2. Predictors of Leukocyte Telomere Length (LTL) at Initial and Annual Assessment 

Table 4 illustrates the results of the investigation for potential predictors of LTL by employing a standard forward, multiple stepwise linear regression model.

***Metabolic syndrome parameters:*** among metabolic syndrome parameters at initial assessment (SBP, waist circumference, Cholesterol, Triglycerides, HDL), waist circumference at initial assessment was the best negative predictor of LTL both at initial (β: −0.14) and annual (β: −0.13) assessment. No significant predictor of Delta LTL was found.

***Body composition parameters:*** among body composition measurements at initial assessment (fat percentage, fat mass, muscle mass, bone mass, fat free mass, total body water and BMR), BMR at initial assessment was the best negative predictor of LTL both at initial (β: −0.54) and annual (β: −0.49) assessment, while bone mass at initial assessment was the best positive predictor of LTL both at initial (β: 0.43) and annual (β: 0.38) assessment. No significant predictor of Delta LTL was found.

***Anthropometric parameters:*** among anthropometric parameters at initial assessment (Weight, Height, BMI, Waist, Hip circumference, Waist to Hip ratio and Waist to height ratio), waist circumference at initial assessment was the best negative predictor of LTL at initial assessment (β: −0.27). Waist to hip ratio at initial assessment was the best negative predictor of LTL at annual assessment (β: −0.15). Waist to height ratio at initial assessment was the best positive predictor of Delta LTL (β: 0.41)**.**

***Glucose metabolism and insulin sensitivity parameters:*** among glucose metabolism and insulin sensitivity parameters (Glucose, Insulin and HbA1C, HOMA, QUICKI), glucose at initial assessment was the best negative predictor of LTL at initial assessment (β: −0.39), followed by QUICKI (β: −0.3) and insulin (β: −1.1). Glucose concentration at initial assessment were the best and sole negative predictor (β: −0.19) of LTL at the annual assessment. No significant predictor of Delta LTL was found. 

### 3.3. Clinical Characteristics, Body Composition and Hematologic, Biochemical and Endocrinologic Parameters

Table 1 illustrates the clinical characteristics and anthropometric parameters (1A), body composition parameters (1B), as well as hematologic investigations (1C), biochemical investigations (1D), cardiometabolic risk factors (1E), endocrinologic investigations (1F), and leucocyte telomere length measurements (1G) in all participants with “good compliance” (*n* = 407) at the initial and annual assessment. Participants were classified as obese, overweight, or of normal-BMI according to the IOTF criteria and their BMI status at initial assessment and the three groups were followed prospectively at 12 months. The statistical comparisons among all variables are also reported in Table 1**.** Children and adolescents with obesity at the initial assessment had significantly higher body weight, BMI, BMI z-score, waist circumference, hip circumference, waist to height ratio, fat percentage, fat mass, basal metabolic rate (BMR), T3 and insulin concentrations, and lower SHBG concentrations and HOMA-IR index than their overweight and normal-BMI counterparts both at the initial and annual assessment. In addition, participants with obesity at the initial assessment had significantly higher ALT, triglycerides, and ApoB concentrations than overweight and normal-BMI participants at the initial assessment and this difference disappeared at the annual assessment. Children and adolescents with obesity at the initial assessment had significantly higher γ-GT and QUICKI index than overweight and normal-BMI participants at the initial assessment and this difference disappeared for overweight participants in the annual assessment. In all three groups of participants, muscle, bone and fat free mass, total body water and BMR, and creatinine, HbA1, IGF-I and FSH concentrations increased significantly at the annual assessment. In subjects with obesity WBC, PLT, folic acid, AST, ALT, γ-GT, LDL, ApoB, HbA1c, and T3 concentrations decreased significantly at the end of the study.

Table 2 illustrates the clinical characteristics and anthropometric parameters (2A), body composition parameters (2B), as well as hematologic investigations (2C), biochemical investigations (2D), cardiometabolic risk factors (2E), endocrinologic investigations (2F), and leucocyte telomere length measurements (2G) in all participants with “good compliance” (*n* = 407), taken as one group at initial and annual assessment, and their statistical comparisons. Following 1 year of participating in the comprehensive, multidisciplinary, personalized, lifestyle intervention program of healthy diet and physical exercise, on average participants demonstrated a significant decrease in their BMI, BMI z-score, waist to height ratio, fat percentage, white blood cells, platelets, ferritin, AST, ALT, γ-GT, LDL-cholesterol, ApoB, Lp(a), HbA1C concentrations, and QUICKI index (*p* < 0.05). In addition, they demonstrated a significant increase in muscle mass, bone mass, fat-free mass, as well as in HDL and ApoA1 concentrations (*p* < 0.05).

## 4. Discussion

In the present study we investigated the effect of a structured, comprehensive, multidisciplinary, personalized lifestyle intervention program of healthy diet and physical exercise on leukocyte telomere length (LTL) in children and adolescents with obesity, overweight, and normal BMI. We found that LTL increased significantly after 1 year of the lifestyle interventions, irrespective of gender, pubertal status or BMI, and was greater in girls than in boys, irrespective of initial pubertal staging, both at the initial and the annual assessment, in participants that demonstrated good compliance in our interventions follow-up. This is of particular importance given the established in the literature age dependent LTL shortening [6]. The implementation of the lifestyle interventions for 1 year, particularly given the high rate of good compliance, also resulted in a significant improvement in clinical (BMI, BMI z-score and waist to height ratio) and body composition indices of obesity (fat percentage, muscle mass, fat free mass, and total body water), inflammatory markers (WBC, platelets, ferritin), hepatic enzymes (AST, ALT, γ-GT), HbA1C, QUICKI index, and lipid profile (HDL, LDL, ApoA1, ApoB) in all participants. These findings indicate that the increased LTL may be associated with a more favorable metabolic profile and decreased morbidity later in life. The main limitation of this prospective intervention study is the lack of a randomized control group without intervention, since the participants were enrolled following a visit on an Outpatient Clinic and entered our multidisciplinary management program.

To the best of our knowledge this is the second study to assess prospectively the changes in LTL in children and adolescents, and the only one with 12 months’ duration. In the previous EVASYON study, Garcia–Calzon et al. showed that a weight loss intervention over 2 months is accompanied by a significant increase in LTL in overweight and obese adolescents [16]. LTL is influenced by changes in the activity of telomerase (telomere terminal transferase), as well as by the alternative lengthening of telomeres pathway [27]. In somatic cells, telomerase activity is very low, almost undetectable, so telomeres shorten progressively with cell division, ultimately leading to loss of telomere protection and a DNA damage response that induces senescence or cell death [27]. Telomere shortening contributes to the rise in mortality rates from multiple diseases typically seen with ageing [28]. It was suggested that telomere attrition is likely a modifiable factor as there is substantial variability in the rate of telomere shortening that is independent of chronological age [6]. Individual risk factors analysis has shown that telomere length variability may be partially explained by lifestyle practices, with healthy lifestyle being associated with longer LTL [29].

We also demonstrated that LTL increased significantly following the 1 year-intervention program both in the subjects that were successful and those who were not successful at losing weight. This is a major finding indicating that lifestyle interventions can potentially be beneficial, despite apparent lack of success at decreasing the BMI. Previous studies showed a relation between LTL elongation and weight loss either with adherence to Mediterranean diet or bariatric surgery [16,30]. The mechanism responsible for LTL elongation is thought to be the active redistribution of younger naive cells regardless of cell types with longer telomeres in the periphery [2]. Younger cells in the circulation were shown to positively influence longevity in experimental animals, via an unknown mechanism [31]. In our study, following the one-year of multidisciplinary, personalized lifestyle intervention program of healthy diet and physical exercise, we showed a significant improvement in clinical (BMI, BMI z-score and waist to height ratio) and body composition indices of obesity (fat percentage, muscle mass, fat free mass, and total body water), inflammatory markers (WBC, platelets, ferritin), hepatic enzymes (AST, ALT, γ-GT), HbA1C, QUICKI index, and lipid profile (HDL, LDL, ApoA1, ApoB) in all participants. In subjects with obesity at the initial assessment there was also a decrease in HbA1C concentrations at the end of the study. These findings are significant given that metabolic abnormalities are likely to predispose to atherosclerotic cardiovascular disease in adult life. They may also indicate that the increased LTL may be associated with a more favorable prognosis and reduced morbidity and mortality later in life [32,33,34,35,36].

It is important to note that LTL did not differ among obese, overweight, and normal-BMI participants at the initial assessment. Although several previous studies showed a negative association between LTL and BMI [15,16,17,18,37], others showed no association between telomere length and adiposity [19,38]. This may be due to the different methodology used in previous studies, where the association was assessed via correlation analysis, while in the present study a direct criterion of adiposity, the IOTF criteria was employed to categorize participants at the initial assessment as obese, overweight, and normal-BMI, followed by repeated measures of ANOVA analysis to compare the three groups [39]. However, when we used forward stepwise regression analysis to evaluate the effect of potential metabolic syndrome risk factors and anthropometric parameters, we showed that waist circumference, a major marker of adiposity, was the best negative predictor of LTL at the initial assessment. Also, the waist to hip ratio at the initial assessment was the best negative predictor of LTL at the annual assessment, while waist to height ratio was the best positive predictor of the change of LTL. This is accompanied by the significant decrease of waist to height ratio after one year of intervention. Reduction of waist to height ratio in obese and overweight participants is significant given that this is an index of central adiposity, which is positively associated with the development of metabolic syndrome and cardiovascular disease [40]. Waist to height ratio is a stronger indicator of cardiovascular disease risk than waist circumference, waist to hip ratio and BMI, making it the most useful among the anthropometric indices and of higher accuracy screening tool for predicting the cardiometabolic risk in childhood, adolescence, and adulthood [41,42,43,44]. Furthermore, the waist to height ratio was associated with pro-oxidation in pre- and early pubertal boys, providing a mechanistic link to the potential benefits of assessing this marker [45].

It is also important to note that glucose concentrations at initial assessment were the best negative predictor of LTL at initial and annual assessment, followed by QUICKI index and insulin concentrations only at initial assessment. Furthermore, obese participants had significantly higher insulin concentrations, and lower SHBG concentrations and HOMA-IR index than overweight and normal-BMI participants both at the initial and annual assessment, while HbA1c concentrations decreased only in obese individuals. Of note, glucose concentrations were the best negative predictor of LTL at 12 months. These findings indicate that glucose homeostasis and insulin resistance are parts of an ongoing process that would potentially require longer term intervention. Consumption of sweetened drinks was associated with shorter LTL length even at a young age [46]. Insulin resistance represents not only an important target for the prevention of diabetes mellitus type 2 and the progressive ‘burn-out’ of β-cell function associated with advanced disease, but also directly contributes to microvascular damage in a pathway-selective manner that is associated with increased oxidative stress. [47]. Increased oxidative stress was associated with adverse clinical outcomes, even in the absence of diabetes, while the process of LTL shortening is accelerated through exposure to oxidative stress and inflammation [46]. Also it was reported that obesity in pre- and early puberty is associated with increased oxidative stress, as shown by the increased markers of pro-oxidation and decreased markers of anti-oxidation [45,48]. In the present study, when assessing the effect of body composition parameters analysis by a forward stepwise multiple regression model, basal metabolic rate (BMR) was the best negative predictor and bone mass was the best positive predictor of LTL at initial assessment. It was shown that short LTL may reflect an underlying mechanism illustrating chronic subsequent deterioration of a person’s metabolic health [49]. At the same time, a negative association between resting metabolic rate and lifespan is the cornerstone of the rate of living and free-radical damage theories of aging [50]. Resting metabolic rate is affected by body mass in experimental animals, while the role of adipose tissue and the understanding of the adipocyte as an endocrine organ is leading us to new insights into obesity and health [51]. In the present study, T3 concentrations where higher in obese than overweight or normal-BMI children and adolescents, and decreased in obese and overweight subjects following the interventions. Increased T3 concentrations (and TSH) in obesity are a signal, part of an adaptive process, aiming to increase BMR and energy consumption in an effort to maintain body weight by regaining a zero energy balance [52]. The positive association between bone mass and LTL in this study was previously described in women with decrease in BMD and the presence of osteoporosis, implicating LTL as a marker of biological aging of the bone [53]. Of particular interest is the fact that LTL was greater in girls than in boys, irrespective of initial pubertal staging, both at initial and annual assessment, while the LTL length was greater in overweight and obese girls than the respective boys. This is an interesting finding, and it corroborates previous studies that showed greater LTL in women and adolescent girls, while no difference is found in the newborn period [7,19,20,54,55,56]. The sexual dimorphism in human longevity was a long standing challenge for science and most likely reflects genomic adaptations that conceptually commence at a very young age [21].

It appears that a structured, comprehensive, multidisciplinary, personalized lifestyle intervention program of healthy diet and physical exercise for 1 year may lead to a significant increase in LTL in children and adolescents, irrespective of gender, pubertal status or BMI, as well as a significant improvement in metabolic syndrome parameters, including clinical and body composition indices of obesity, inflammatory markers, hepatic enzymes, markers of insulin resistance and lipid profile. These findings highlight the importance of adopting a healthy lifestyle in preventing metabolic abnormalities and reducing the risk of atherosclerotic cardiovascular disease later in life. Given that LTL increased in both the successful and not successful at weight loss participants, the benefits of implementing a lifestyle intervention program are self-evident. Our findings also provide a conceptual link between LTL, a well-studied marker of longevity, and the risk of cardiovascular disease, which may arise in childhood. Finally, our study provides evidence that sexual dimorphism in human longevity is evident at an early age. Further studies describing a randomized experiment in which LTL is compared between a treatment and nonintervention control group are required to delineate the underlying mechanisms responsible for the increased LTL in subjects with an improvement in cardiometabolic profile.

## Figures and Tables

**Table 1:** Clinical characteristics and anthropometric parameters (1A), body composition parameters (1B), hematology (1C), biochemistry (1D), cardiometabolic (CVD) risk factors (1E), hormonal (1F) and leucocyte telomere length (LTL) (1G) measurements in all subjects with “good compliance” (*n* = 407) at initial and annual assessment.

**A. Anthropometry**	**Initial assessment**				**Annual assessment**				**P_between timepoints_**
	**Obese**	**Overweight**	**Normal BMI**	**P_within baseline_**	**Obese**	**Overweight**	**Normal BMI**	**P_within follow-up_**	
Age (years)	10.04 ± 0.20	10.48 ± 0.20	9.65 ± 0.39	NS	11.04 ± 0.22 *	11.52 ± 0.21 *	10.74 ± 0.44 *	NS	0.01/0.01/0.01
Body weight (kg)	62.54 ± 1.42	52.64 ± 1.21 ^#^	38.61 ± 1.58 ^+,#^	0.01	64.06 ± 1.56 *	55.13 ± 1.35 *^,#^	43.84 ± 1.72 *^,+,#^	0.01	0.01/0.01/0.01
Height (cm)	144.47 ± 1.17	145.43 ± 1.08	138.03 ± 2.02	NS	149.77 ± 1.26 *	151.37 ± 1.14 *	147.06 ± 2.06 *	NS	0.01/0.01/0.01
BMI (kg/m^2^)	28.62 ± 0.28	24.21 ± 0.21 ^#^	19.54 ± 0.35 ^+,#^	0.01	27.15 ± 0.32 *	23.58 ± 0.27 *^,#^	19.81 ± 0.38 ^+,#^	0.01	0.01/0.01/NS
BMI z-score	3.22 ± 0.07	1.53 ± 0.02 ^#^	0.14 ± 0.10 ^+,#^	0.01	2.41 ± 0.07 *	1.18 ± 0.06 *^,#^	0.25 ± 0.08 ^+,#^	0.01	0.01/0.01/NS
SBP (mmHg)	114.10 ± 0.96	110.76 ± 0.83	104.48 ± 1.32 ^+^	0.05	114.26 ± 1.06	109.64 ± 1.17 ^#^	106.65 ± 2.12 ^#^	0.01	NS/NS/NS
DBP (mmHg)	67.02 ± 0.78	65.75 ± 0.78	61.00 ± 1.17 ^+,#^	0.01	68.41 ± 0.84	65.54 ± 0.82	63.70 ± 1.58 ^#^	0.05	NS/NS/NS
Waist (cm)	86.73 ± 0.90	78.42 ± 0.80 ^#^	67.49 ± 1.31 ^+,#^	0.01	87.47 ± 1.24	80.05 ± 1.27 ^#^	70.57 ± 2.10 ^+,#^	0.01	NS/NS/NS
Hip (cm)	92.05 ± 1.07	86.60 ± 1.01 ^#^	74.72 ± 1.66 ^+,#^	0.05	94.56 ± 1.36	89.08 ± 1.24 ^#^	81.89 ± 2.07 *^,+,#^	0.01	NS/NS/0.01
Waist to Hip ratio	0.96 ± 0.01	0.91 ± 0.01 ^#^	0.92 ± 0.02	0.05	0.93 ± 0.01	0.90 ± 0.01 ^#^	0.86 ± 0.02 *^,#^	0.01	NS/NS/0.05
Waist to Height ratio	0.60 ± 0.01	0.54 ± 0.01 ^#^	0.49 ± 0.01 ^+,#^	0.01	0.58 ± 0.01 *	0.53 ± 0.01 ^#^	0.48 ± 0.01 ^+,#^	0.01	0.01/NS/NS
**B. Body composition**	**Initial assessment**				**Annual assessment**				**P_between timepoints_**
	**Obese**	**Overweight**	**Normal BMI**	**P_within baseline_**	**Obese**	**Overweight**	**Normal BMI**	**P_within follow-up_**	
Fat Percentage (%)	37.44 ± 0.38	32.23 ± 0.34 ^#^	25.6 ± 0.74 ^+,#^	0.01	35.49 ± 0.42 *	31.09 ± 0.39 *^,#^	25.02 ± 0.75 ^+,#^	0.01	0.01/0.01/NS
Fat Mass (kg)	24.15 ± 0.69	17.17 ± 0.51 ^#^	10.37 ± 0.58 ^+,#^	0.01	23.76 ± 0.70	17.76 ± 0.57 ^#^	10.89 ± 0.60 ^+,#^	0.01	NS/NS/NS
Muscle Mass (kg)	36.77 ± 0.79	33.63 ± 0.73	27.55 ± 1.09 ^+,#^	0.01	39.48 ± 0.83 *	36.80 ± 0.75 *^,#^	29.94 ± 1.10 *^,+,#^	0.01	0.01/0.01/0.01
Bone mass (kg)	1.99 ± 0.04	1.85 ± 0.04	1.54 ± 0.06 ^+,#^	0.01	2.13 ± 0.04 *	2.00 ± 0.04 *^,#^	1.66 ± 0.06 *^,+,#^	0.01	0.01/0.01/0.01
Fat Free Mass (kg)	38.76 ± 0.83	35.47 ± 0.77	29.09 ± 1.15 ^+,#^	0.01	41.64 ± 0.88 *	38.80 ± 0.79 *^,#^	31.61 ± 1.15 *^,+,#^	0.01	0.01/0.01/0.01
Total Body Water (kg)	28.38 ± 0.61	25.97 ± 0.56	21.29 ± 0.84 ^+,#^	0.01	30.46 ± 0.64 *	28.40 ± 0.58 *^,#^	23.15 ± 0.84 *^,+,#^	0.01	0.01/0.01/0.01
BMR (kJ)	6491.92 ± 90.37	6027.06 ± 83.21 ^#^	5287.79 ± 102.68 ^+,#^	0.01	6693.43 ± 95.96 *	6281.82 ± 84.99 *^,#^	5464.08 ± 105.64 *^,+,#^	0.01	0.01/0.01/0.01
**C. Hematology**	**Initial assessment**				**Annual assessment**				**P_between timepoints_**
	**Obese**	**Overweight**	**Normal BMI**	**P_within baseline_**	**Obese**	**Overweight**	**Normal BMI**	**P_within follow-up_**	
WBC	7.74 ± 0.13	7.07 ± 0.15 ^#^	7.12 ± 0.27 ^#^	0.05	7.54 ± 0.14 *	7.16 ± 0.17	6.45 ± 0.26 *^,+,#^	0.01	0.05/NS/0.05
RBC	5.02 ± 0.05	5.06 ± 0.06	4.93 ± 0.07	NS	5.20 ± 0.20	5.05 ± 0.04	4.99 ± 0.08	NS	NS/NS/NS
Hb	12.97 ± 0.14	13.08 ± 0.19	12.75 ± 0.12	NS	12.94 ± 0.08	13.19 ± 0.09 *^,#^	12.93 ± 0.15	0.05	NS/0.01/NS
Hct	40.49 ± 0.23	40.81 ± 0.32	40.15 ± 0.35	NS	40.56 ± 0.22	40.96 ± 0.25	40.29 ± 0.41	NS	NS/NS/NS
PLT	303.93 ± 4.20	292.74 ± 5.06 ^#^	297.03 ± 7.61	0.05	288.30 ± 5.27 *	290.29 ± 4.81	275.93 ± 6.45 *	NS	0.01/NS/0.05
ESR	19.34 ± 0.92	18.35 ± 1.01	12.38 ± 1.25 ^+,#^	0.05	18.65 ± 0.94	15.93 ± 0.98	13.75 ± 1.53 ^#^	0.01	NS/NS/NS
Ferritin	61.71 ± 2.89	56.90 ± 2.28	51.07 ± 3.26	NS	56.45 ± 2.37 *	52.85 ± 2.80	48.08 ± 4.62 *	NS	0.05/NS/0.05
Folic Acid	11.75 ± 0.39	12.79 ± 0.84	13.60 ± 1.54 ^#^	0.05	9.40 ± 0.49 *	9.78 ± 0.43 *	12.87 ± 1.65 ^#^	0.05	0.01/0.01/NS
**D. Biochemistry**	**Initial assessment**				**Annual assessment**				**P_between timepoints_**
	**Obese**	**Overweight**	**Normal BMI**	**P_within baseline_**	**Obese**	**Overweight**	**Normal BMI**	**P_within follow-up_**	
Urea	28.84 ± 0.42	28.59 ± 0.46	28.30 ± 0.88	NS	28.59 ± 0.46	28.56 ± 0.55	26.71 ± 0.94	NS	NS/NS/NS
Creatinine	0.49 ± 0.01	0.50 ± 0.01	0.46 ± 0.02	NS	0.52 ± 0.01 *	0.52 ± 0.01 *	0.52 ± 0.02 *	NS	0.01/0.01/0.01
Uric Acid	7.53 ± 1.01	5.22 ± 0.58	5.97 ± 1.95	NS	4.88 ± 0.08	5.05 ± 0.45	6.72 ± 1.80	NS	NS/NS/NS
K	4.44 ± 0.02	4.37 ± 0.03	4.37 ± 0.05	NS	4.39 ± 0.84	4.43 ± 0.03	4.45 ± 0.07	NS	NS/NS/NS
Na	137.07 ± 1.33	138.60 ± 1.16	138.13 ± 2.27	NS	138.99 ± 1.00	139.04 ± 1.07	133.02 ± 4.60	NS	NS/NS/NS
Cl	103.76 ± 0.68	102.76 ± 0.55	104.66 ± 1.69	NS	101.03 ± 1.55	100.47 ± 2.39	107.00 ± 3.53	NS	NS/NS/NS
AST	23.88 ± 0.42	23.07 ± 0.45	23.33 ± 0.49	NS	22.05 ± 0.43 *	22.33 ± 0.52 *	24.19 ± 1.02 ^#^	0.05	0.01/0.05/NS
ALT	22.36 ± 0.85	19.35 ± 0.71 ^#^	15.78 ± 0.71 ^+,#^	0.05	19.52 ± 0.60 *	18.62 ± 0.72	17.52 ± 1.06	NS	0.01/NS/NS
γ-GT	14.97 ± 0.42	13.19 ± 0.34 ^#^	11.08 ± 0.38 ^#^	0.01	13.66 ± 0.35 *	13.54 ± 0.45	11.21 ± 0.43 ^+,#^	0.05	0.01/NS/NS
Albumin	4.70 ± 0.03	4.72 ± 0.03	4.67 ± 0.07	NS	4.58 ± 0.02 *	4.64 ± 0.03	4.68 ± 0.08	NS	0.01/NS/NS
ALP	233.35 ± 5.49	231.02 ± 6.09	230.13 ± 10.15	NS	218.56 ± 5.67 *	220.84 ± 7.09 *	224.71 ± 14.56	NS	0.01/0.05/NS
PO4	4.77 ± 0.06	4.68 ± 0.04	5.39 ± 0.72	NS	4.94 ± 0.28	4.72 ± 0.04	4.63 ± 0.08	NS	NS/NS/NS
Ca	10.23 ± 0.38	9.83 ± 0.03	9.78 ± 0.04	NS	9.79 ± 0.02 *	9.83 ± 0.03	9.74 ± 0.07	NS	0.01/NS/NS
**E. CVD risk factors**	**Initial assessment**				**Annual assessment**				**P_between timepoints_**
	**Obese**	**Overweight**	**Normal BMI**	**P_within baseline_**	**Obese**	**Overweight**	**Normal BMI**	**P_within follow-up_**	
Cholesterol	152.27 ± 2.40	154.90 ± 2.46	151.63 ± 4.60	NS	154.75 ± 2.20	155.45 ± 2.89	147.91 ± 6.33	NS	NS/NS/NS
Triglycerides	84.82 ± 2.68	69.74 ± 2.54 ^#^	69.40 ± 5.18 ^#^	0.05	82.74 ± 3.07	74.75 ± 3.40 *	71.31 ± 5.97	NS	NS/0.05/NS
HDL	50.58 ± 0.87	53.46 ± 1.03	57.60 ± 1.68 ^#^	0.05	51.79 ± 0.96	55.81 ± 1.12 *^,#^	59.48 ± 2.37 *^,#^	0.01	NS/0.01/0.05
LDL	89.57 ± 1.59	89.72 ± 1.95	82.95 ± 3.44	NS	88.63 ± 1.67 *	87.02 ± 2.05	80.62 ± 2.90	NS	0.05/NS/NS
ApoA1	137.02 ± 1.66	139.80 ± 1.54	146.17 ± 3.66	NS	140.60 ± 1.69	143.59 ± 1.83	150.60 ± 3.32 ^#^	0.05	NS/NS/NS
ApoB	75.19 ± 1.20	72.34 ± 1.26 ^#^	68.03 ± 1.97 ^#^	0.01	72.61 ± 1.14 *	70.05 ± 1.45	66.05 ± 2.11 ^#^	0.05	0.01/NS/NS
Lp(a)	15.93 ± 1.47	15.27 ± 1.66	14.96 ± 2.66	NS	16.62 ± 1.80	14.38 ± 2.03	10.23 ± 1.92	NS	NS/NS/NS
Glucose	79.03 ± 0.57	78.35 ± 0.76	78.02 ± 1.00	NS	80.91 ± 0.47 *	81.00 ± 0.64 *	79.76 ± 0.99	NS	0.01/0.01/NS
HbA1c	5.28 ± 0.02	5.22 ± 0.02	5.18 ± 0.03	NS	5.22 ± 0.02 *	5.20 ± 0.02	5.19 ± 0.0	NS	<0.01/NS/NS
HbA1	5.98 ± 0.03	5.91 ± 0.03 ^#^	5.93 ± 0.05	0.01	6.02 ± 0.03 *	5.94 ± 0.03 *	5.98 ± 0.05 *	NS	0.05/0.01/0.05
HOMA-IR	3.62 ± 0.17	2.55 ± 0.12 ^#^	1.83 ± 0.16 ^#^	0.05	3.47 ± 0.17	2.91 ± 0.14 ^#^	1.98 ± 0.16 ^+,#^	0.01	NS/NS/NS
QUICKI	0.33 ± 0.01	0.34 ± 0.01 ^#^	0.37 ± 0.01 ^+,#^	0.01	0.33 ± 0.01	0.33 ± 0.01 *	0.35 ± 0.01 *^,+,#^	0.05	NS/0.01/0.05
**F. Hormones**	**Initial assessment**				**Annual assessment**				**P_between timepoints_**
	**Obese**	**Overweight**	**Normal BMI**	**P_within baseline_**	**Obese**	**Overweight**	**Normal BMI**	**P_within follow-up_**	
TSH	2.92 ± 0.09	2.93 ± 0.13	2.82 ± 0.20	NS	3.04 ± 0.12	2.94 ± 0.15	2.39 ± 0.17 ^+,#^	0.05	NS/NS/NS
FT4	1.76 ± 0.55	1.13 ± 0.01	1.31 ± 0.16	NS	1.10 ± 0.01	1.10 ± 0.01	1.09 ± 0.03	NS	NS/NS/NS
T3	148.68 ± 1.71	138.40 ± 2.15 ^#^	134.98 ± 3.59 ^#^	0.01	141.90 ± 2.23 *	135.51 ± 2.61 *^,#^	131.13 ± 4.00 ^#^	0.05	0.01/0.05/NS
AntiTG	21.14 ± 0.66	22.86 ± 1.58	22.89 ± 1.95	NS	27.72 ± 5.56	26.51 ± 3.66	30.08 ± 7.54	NS	NS/NS/NS
AntiTPO	17.11 ± 4.13	30.23 ± 8.75	30.12 ± 16.22	NS	18.04 ± 5.11	32.10 ± 10.97	35.26 ± 22.98	NS	NS/NS/NS
IGF1	304.23 ± 10.27	322.79 ± 14.29	269.96 ± 20.79	NS	387.42 ± 14.66 *	394.87 ± 16.93 *	336.14 ± 22.18 *	NS	0.01/0.01/0.01
IGFBP3	5.03 ± 0.07	5.11 ± 0.08	4.73 ± 0.15	NS	6.07 ± 0.68	5.35 ± 0.11	5.01 ± 0.15	NS	NS/NS/NS
PRL	11.29 ± 0.38	11.63 ± 0.51	11.73 ± 0.94	NS	12.51 ± 0.52 *	12.47 ± 0.56 *	12.53 ± 0.86	NS	0.01/0.01/NS
LH	1.94 ± 0.19	2.92 ± 0.62	1.19 ± 0.22	NS	2.91 ± 0.26 *	3.39 ± 0.40	2.74 ± 0.52	NS	<0.01/NS/NS
FSH	2.53 ± 0.12	2.86 ± 0.19	2.29 ± 0.22	NS	3.00 ± 0.15 *	3.61 ± 0.26 *^,#^	3.42 ± 0.37 *	0.05	0.01/0.01/0.05
ACTH	34.35 ± 5.45	28.60 ± 1.81	22.14 ± 1.41	NS	29.67 ± 1.62	28.72 ± 1.82	25.20 ± 2.07	NS	NS/NS/NS
Cortisol	13.53 ± 0.40	14.17 ± 0.49	15.48 ± 2.31 ^+,#^	0.05	13.30 ± 0.44	13.41 ± 0.48	11.39 ± 0.60 *	NS	NS/NS/0.01
PTH	33.41 ± 1.88	33.22 ± 0.87	35.90 ± 0.90	NS	35.58 ± 2.06	34.55 ± 0.81	36.51 ± 0.91	NS	NS/NS/NS
25OHVitD	24.29 ± 1.38	24.10 ± 0.73	25.74 ± 1.23	NS	23.81 ± 0.73	26.35 ± 2.32	28.23 ± 1.91	NS	NS/NS/NS
Insulin	18.24 ± 0.80	12.91 ± 0.57 ^#^	9.30 ± 0.76 ^+,#^	0.01	17.22 ± 0.82	14.48 ± 0.68 ^#^	9.81 ± 0.73 ^+,#^	0.01	NS/NS/NS
SHBG	43.18 ± 1.81	53.47 ± 2.49 ^#^	80.23 ± 5.79 ^+,#^	0.01	44.62 ± 2.09	52.87 ± 2.43 ^#^	73.41 ± 6.59 ^+,#^	0.01	NS/NS/NS
**G. LTL**	**Initial assessment**				**Annual assessment**				**P_between timepoints_**
	**Obese**	**Overweight**	**Normal BMI**	**P_within baseline_**	**Obese**	**Overweight**	**Normal BMI**	**P_within follow-up_**	
LTL	1.35 ± 0.02	1.36 ± 0.02	1.33 ± 0.03	NS	1.41 ± 0.02 *	1.42 ± 0.02 *	1.38 ± 0.03 *	NS	0.01/0.01/0.01

All results are presented as mean ± SE. Subjects were classified as obese, overweight, or with normal BMI according to IOTF criteria at initial assessment. Tables present comparisons among three groups at both initial and annual assessment. All measured variables were compared by employing repeated-measures ANOVA. Significant main effects were revealed by the LSD posthoc test. Statistical significance was set at (*p* < 0.05, rounded to 0.05 in Table), while strong significance (*p* < 0.01, rounded to 0.01 in Table) is also noted. NS: nonsignificant (*p* > 0.05) difference. *: Indicates significant difference between initial and annual assessment, timepoints respectively. ^+^: Indicates significant difference from Overweight group. ^#^: Indicates significant difference from Obese group. *p*-values between two timepoints refer to obese, overweight, and normal BMI respectively.

**Table 2:** Clinical characteristics and anthropometric parameters (2A), body composition parameters (2B), hematology (2C), biochemistry (2D), cardiometabolic (CVD) risk factors (2E), hormonal (2F) and leucocyte telomere length (LTL) (2G) measurements in all subjects with “good compliance” (*n* = 407), at initial and annual assessment.

**A. anthropometry**	**Initial assessment**	**Annual assessment**	**P_between timepoints_**
Age (years)	10.14 ± 0.13	11.23 ± 0,15	<0.01
Body weight (kg)	55.99 ± 0.95	58.61 ± 1.03 *	<0.01
Height (cm)	143.95 ± 0.77	150.02 ± 0.82 *	<0.01
BMI (kg/m^2^)	25.91 ± 0.22	25.06 ± 0.24 *	<0.01
BMI z-score	2.25 ± 0.06	1.72 ± 0.06 *	<0.01
SBP (mmHg)	111.62 ± 0.62	112.05 ± 0.77	NS
DBP (mmHg)	65.75 ± 0.52	67.04 ± 0.58	NS
Waist (cm)	81.41 ± 0.65	83.37 ± 0.92	NS
Hip (cm)	87.95 ± 0.75	91.51 ± 0.93	NS
Waist to Hip ratio	0.94 ± 0.01	0.91 ± 0.01	NS
Waist to Height ratio	0.57 ± 0.01	0.55 ± 0.01 *	<0.01
**B. Body composition**	**Initial assessment**	**Annual assessment**	**P_between timepoints_**
Fat Percentage (%)	34.09 ± 0.31	32.57 ± 0.32 *	<0.01
Fat Mass (kg)	19.92 ± 0.46	19.94 ± 0.47	NS
Muscle Mass (kg)	34.45 ± 0.52	37.23 ± 0.32 *	<0.01
Bone mass (kg)	1.88 ± 0.03	2.02 ± 0.03 *	<0.01
Fat Free Mass (kg)	36.33 ± 0.55	37.23 ± 0.54 *	<0.01
Total Body Water (kg)	26.60 ± 0.40	28.73 ± 0.42 *	<0.01
BMR (kJ)	6171.22 ± 59.48	6382.51 ± 62.21	NS
**C. Hematology**	**Initial assessment**	**Annual assessment**	**P_between timepoints_**
WBC	7.43 ± 0.09	7.28 ± 0.10 *	<0.05
RBC	5.02 ± 0.03	5.12 ± 0.11	NS
Hb	12.88 ± 0.10	13.03 ± 0.06 *	<0.01
Hct	40.56 ± 0.17	40.67 ± 0.16	NS
PLT	299.21 ± 2.99	287.54 ± 3.37 *	<0.01
ESR	18.18 ± 0.63	17.14 ± 0.64	NS
Ferritin	58.66 ± 1.77	54.29 ± 1.69 *	<0.01
Folic Acid	13.17 ± 0.41	9.88 ± 0.36 *	<0.01
**D. Biochemistry**	**Initial assessment**	**Annual assessment**	**P_between timepoints_**
Urea	28.69 ± 0.29	28.36 ± 0.33	NS
Creatinine	0.49 ± 0.01	0.52 ± 0.01 *	<0.01
Uric Acid	5.45 ± 0.62	5.15 ± 0.26	NS
K	4.41 ± 0.02	4.40 ± 0.05	NS
Na	137.73 ± 0.86	138.32 ± 0.84	NS
Cl	103.57 ± 0.47	101.53 ± 1.24	NS
AST	23.54 ± 0.28	22.39 ± 0.32 *	<0.01
ALT	20.50 ± 0.53	18.98 ± 0.43 *	<0.01
γGT	13.86 ± 0.26	13.34 ± 0.25 *	<0.01
Albumin	4.71 ± 0.02	4.61 ± 0.02 *	<0.01
ALP	232.13 ± 3.80	220.07 ± 4.25 *	<0.01
PO4	4.82 ± 0.10	4.83 ± 0.15	NS
Ca	10.04 ± 0.20	9.80 ± 0.02 *	<0.01
**E. CVD risk factors**	**Initial assessment**	**Annual assessment**	**P_between timepoints_**
Cholesterol	153.08 ± 1.64	154.22 ± 1.71	NS
Triglycerides	77.72 ± 1.82	78.64 ± 2.15	NS
HDL	52.46 ± 0.63	54.07 ± 0.71 *	<0.01
LDL	90.37 ± 1.16	87.15 ± 1.20 *	<0.01
ApoA1	139.14 ± 1.13	142.80 ± 1.18 *	<0.05
ApoB	73.31 ± 0.81	70.98 ± 0.83 *	<0.01
Lp(a)	15.58 ± 1.02	15.12 ± 1.22	NS
Glucose	78.67 ± 0.42	80.81 ± 0.36 *	<0.01
HbA1c	5.25 ± 0.01	5.21 ± 0.01 *	<0.01
HbA1	5.98 ± 0.02	5.99 ± 0.02 *	<0.01
HOMA-IR	3.02 ± 0.11	3.10 ± 0.10	NS
QUICKI	0.34 ± 0.01	0.33 ± 0.01 *	<0.01
**F. Hormones**	**Initial assessment**	**Annual assessment**	**P_between timepoints_**
TSH	2.91 ± 0.07	2.93 ± 0.09	NS
FT4	1.49 ± 0.29	1.61 ± 0.51	NS
T3	143.38 ± 1.28	138.42 ± 1.58 *	<0.01
AntiTG	21.96 ± 0.69	27.57 ± 3.34	NS
AntiTPO	23.37 ± 4.29	24.98 ± 5.42	NS
IGF1	306.18 ± 7.81	384.02 ± 10.17 *	<0.01
IGBP3	5.02 ± 0.05	5.69 ± 0.37	NS
PRL	11.47 ± 0.29	12.50 ± 0.35 *	<0.01
LH	2.18 ± 0.24	3.06 ± 0.21 *	<0.01
FSH	2.62 ± 0.09	3.26 ± 0.13 *	<0.01
ACTH	30.79 ± 2.95	28.82 ± 1.10	NS
Cortisol	14.00 ± 0.40	13.11 ± 0.30 *	<0.01
PTH	34.64 ± 0,61	35.70 ± 0.61	NS
25OHVitD	24.41 ± 0.78	25.24 ± 0.95	NS
Insulin	15.26 ± 0.50	15.40 ± 0.52	NS
SHBG	51.90 ± 1.62	50.97 ± 1.67	NS
**G. LTL**	**Initial assessment**	**Annual assessment**	**P_between timepoints_**
LTL	1.35 ± 0.01	1.41 ± 0.01 *	<0.01

All results are presented as mean ± SE. All measured variables were compared by employing repeated-measures ANOVA. Significant main effects were revealed by the LSD posthoc test. Statistical significance was set at (*p* < 0.05), while strong significance (*p* < 0.01) is also noted. NS: nonsignificant (*p* > 0.05) difference. *: Indicates significant difference between baseline and the 12 months follow-up, timepoint.

**Table 3:** LTL measurements and respective comparisons at baseline and at 12 months follow-up of participants with “good compliance” (*n* = 407), according to criterion of success at weight loss. Subjects were considered successful at weight loss (“YES” group), either if respective BMI z-score decreased more than 0.6 at 12 months follow-up (Criterion A), or if respective IOTF category (normal weight, overweight and obese) improved by one or more categories (Criterion Β). Unsuccessful participants (“NO” group) were participants not fulfilling the respective criteria.

**Criterion A**	**YES (*n* = 193)**		**P_between timepoints_**	**No (*n* = 214)**		**P_between timepoints_**
	**Initial** **assessment**	**Annual assessment**		**Initial** **assessment**	**Annual assessment**	
**LTL**	1.35 ± 0.02	1.41 ± 0.02 *	<0.01	1.37 ± 0.02	1.43 ± 0.02 *	<0.01
**Criterion B**	**YES (*n* = 175)**		**P_between timepoints_**	**No (*n* = 232)**		**P_between timepoints_**
	**Initial** **assessment**	**Annual assessment**		**Initial** **assessment**	**Annual assessment**	
LTL	1.33 ± 0.02	1.40 ± 0.02 *	<0.01	1.38 ± 0.02	1.43 ± 0.02 *	<0.01

All results are presented as mean ± SE. All measured variables were compared by employing repeated-measures ANOVA. Significant main effects were revealed by the LSD post-hoc test. Statistical significance was set at (*p* < 0.05), while strong significance (*p* < 0.01) is also noted. * Indicates significant difference between baseline and the 12 months follow-up, time-points respectively.

**Table 4:** Potential predictors in descending order of LTL at initial assessment, at annual assessment and the respective change (Delta) of LTL–dependent variables. Model A (Table 4A) assessed metabolic syndrome parameters, at initial assessment (SBP, waist circumference, cholesterol, triglycerides, and HDL; independent variables). Model B (Table 4B) assessed body composition parameters, at initial assessment (fat percentage, fat mass, muscle mass, bone mass, fat free mass, total body water and BMR; independent variables). Model C (Table 4C) assessed anthropometric parameters, at initial assessment (weight, height, BMI, waist, hip circumference, waist to hip ratio and waist to height ratio; independent variables). Model D (Table 4D) assessed glucose metabolism and insulin sensitivity parameters, at initial assessment (glucose, insulin, HbA1C, HOMA and QUICKI; independent variables).

	LTL Initial Assessment	LTL Annual Assessment	Delta LTL	*p*
Table 4A				
Waist circumference	β: −0.14	β: −0.13	-	<0.01/<0.05/NS
Table 4B				
BMR	β: −0.54	β: −0.49	-	<0.01/<0.01/NS
Bone mass	β: 0.43	β: 0.13	-	<0.05/<0.05/NS
Table 4C				
Waist circumference	β: −0.27	-	-	<0.01/NS/NS
Waist to hip ratio	-	β: −0.15	-	NS/<0.01/NS
Waist to height ratio	-	-	β: 0.41	NS/NS/<0.05
Table 4D				
Glucose	β: −0.39	β: −0.19	-	<0.01/0.01/NS
QUICKI	β: −0.3	-	-	<0.05/NS/NS
Insulin	β: −1.1	-	-	<0.05/NS/NS

Standard forward, stepwise multiple regression models were employed, and beta (β) coefficients are reported. Statistical significance was set at (*p* < 0.05), while strong significance (*p* < 0.01) is also noted. NS: Non-significant (*p* > 0.05) difference.

## Data Availability

The data presented in this study are available on request from the corresponding author. The data are not publicly available due to privacy restrictions.
